# High-Entropy Sulfide Nanoarchitectures with Triple-Shelled Hollow Design for Durable Sodium–Ion Batteries

**DOI:** 10.3390/nano15120881

**Published:** 2025-06-07

**Authors:** Mingyang Chen, Yan Liu, Zhenchun Fang, Yinan Wang, Shaonan Gu, Guowei Zhou

**Affiliations:** 1 Key Laboratory of Fine Chemicals in Universities of Shandong, Jinan Engineering Laboratory for Multi-Scale Functional Materials, School of Chemistry and Chemical Engineering, Qilu University of Technology (Shandong Academy of Sciences), Jinan 250353, China; 2 Zaozhuang Vocational College of Science & Technology, Tengzhou 277500, China

**Keywords:** sodium–ion batteries, hollow multi-shelled structures, high-entropy materials, transition metal sulfides

## Abstract

Metal sulfides are promising anode candidates for sodium–ion batteries (SIBs) due to their high theoretical capacities. However, their practical application is limited by significant volume extension and sluggish Na^+^ diffusion during cycling, which lead to rapid capacity degradation and poor long-term stability. In this work, we report the rational design of a hollow triple-shelled high-entropy sulfide (NaFeZnCoNiMn)_9_S_8_, synthesized through sequential templating method under hydrothermal conditions. Transmission electron microscopy confirms its well-defined three-shelled architecture. The inter-shell voids effectively buffer Na^+^ insertion/desertion-induced volume extension, while the tailored high-entropy matrix enhances electronic conductivity and accelerates Na^+^ transport. This synergistic design yields outstanding performance, including a high initial Coulombic efficiency (ICE) of 94.1% at 0.1 A g^−1^, low charge-transfer resistance (0.32~2.54 Ω), fast Na^+^ diffusion efficiency (10^−8.5^–10^−10.5^ cm^2^ s^−1^), and reversible capacity of 582.6 mAh g^−1^ after 1600 cycles at 1 A g^−1^ with 91.2% capacity retention. These results demonstrate the potential of high-entropy, multi-shelled architectures as a robust platform for next-generation durable SIB anodes.

## 1. Introduction

With the rapid growth of the global renewable energy sector and increasing emphasis on environmental sustainability, sodium–ion batteries (SIBs) have garnered significant attention for large-scale energy storage and electric transportation applications, owing to their low cost, abundant sodium resources, high safety, and environmental compatibility [1,2,3]. However, the relatively large ionic radius of Na^+^ induces substantial volume changes in electrode materials during repeated Na^+^ insertion/desertion, leading to structural degradation and severely hindering ion transport kinetics [4,5]. As a result, the rational design of both material composition and architecture to mitigate volume expansion and accelerate Na^+^ diffusion has emerged as a central challenge in advancing the electrochemical performance of SIBs [6,7,8].

Transition metal sulfides (TMSs) have been widely explored as anode materials for SIBs due to their high theoretical capacities and abundant redox active sites. Representative systems such as NiS_2_ [9], FeS/SnS_2_ [10], and FeS_x_@SC [11] have exhibited promising electrochemical performance. High-entropy materials featuring the incorporation of multiple metal elements into a single solid-solution lattice enable high-entropy effect and synergistic effect, which not only improve Na^+^ diffusion kinetics but also enhance structural stability during repeated Na^+^ insertion/desertion. More recently, high-entropy sulfides such as (FeCoNiCuRu)S_2_ [12], Na(MnFeCoNi)_1/2_S [13], and (CrFeCoNiMn)_4_S_5_ [14] have demonstrated outstanding capacity retention and significantly reduced charge-transfer resistance.

To date, the dominant method for synthesizing high-entropy materials is mainly high-temperature annealing; however, it typically produces powders or layered structures with limited microstructural precision [15,16]. Although several high-entropy sulfides like HE-FeS_2_/S have demonstrated reversible capacities in the range of 410–560 mAh g^−1^ and rapid capacity fading after 530 cycles due to structural instability [17]. In contrast, hollow multi-shelled structures (HoMSs) could offer significant advantages for long-term electrochemical stability [18,19]. The inter-shell voids effectively buffer the mechanical stress induced by repeated Na^+^ insertion and desertion, while the multi-shelled configuration reduces ion/electron diffusion distances and sequentially minimizes internal resistance. Although various metal sulfide HoMSs, such as hollow Co_9_S_8_/Ni_3_S_4_ composites [20], yolk–shell NiCoMn sulfide spheres [21], and core–shell CoFeS_2_/C hybrids [22], have shown promising capability to enhance SIBs performance, their integration into high entropy with HoMSs remains rare, mainly because of the significant synthetic complexity. Similarly, HoMSs materials demonstrate improved buffering capabilities; however, those lacking compositional complexity, such as Sb@C@TiO_2_, exhibit relatively low reversible capacities, with only 193 mAh g^−1^ at 1 A g^−1^ [23]. Thus, the combination of multi-shelled nanostructures with high-entropy design represents a compelling strategy for next-generation electrode development.

In this work, we present a modified sequential templating strategy wherein carbon microspheres serve as sacrificial scaffolds to adsorb mixed-metal precursors. During hydrothermal treatment, the metal ions coordinate with sucrose and become uniformly distributed within the carbon matrix. Calcination in air removes the carbon template while driving the formation of concentric oxide shells, resulting in a hierarchical oxide precursor with a hollow triple-shell structure. Subsequently, vapor-phase sulfidation converts the Fe_3_O_4_-dominant high-entropy oxide (HEO) into a high-entropy sulfide, with Fe_4.2_Ni_4.8_S_8_ as the primary crystalline phase. Remarkably, despite the integration of multiple transition metal cations, both the precursor oxide and the final sulfide maintain excellent crystallinity and the targeted hollow triple-shell morphology. Notably, the entropy-stabilized phase enhances structural integrity by suppressing phase separation, which complements the stress-buffering capability of the multi-shelled architecture. Together, these features ensure excellent cycling durability under repeated Na^+^ insertion/extraction. The multi-metal charge-compensation effect facilitates fast Na^+^ diffusion and significantly improves cycling stability, delivering a high reversible capacity of 582.6 mAh g^−1^ after 1600 cycles at 1 A g^−1^. These results highlight the synergistic benefits of high-entropy design and multi-shell structuring in advancing SIBs anode materials.

## 2. Results and Discussions

**Scheme 1:**
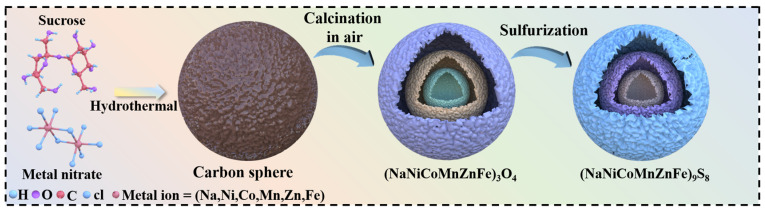
Schematic illustration of the synthesis process and structural configuration of hollow triple-shelled (NaFeZnCoNiMn)_9_S_8_ nanomaterials.

The synthesis of (NaFeZnCoNiMn)_9_S_8_ is depicted in Scheme 1 and comprises a rationally designed multi-step strategy, in which each stage is structurally contributive to the formation of the final hollow multi-shelled architecture. Initially, a homogeneous aqueous solution containing sucrose and metal salts was subjected to hydrothermal treatment. During this process, metal cations coordinated with sucrose molecules and were uniformly embedded into the resulting amorphous carbon microspheres.

Subsequent air calcination played a dual role: it removed the carbon template and oxidized the embedded metal species, leading to the formation of concentric oxide shells. Notably, a radial temperature gradient across the microspheres during calcination induces a competition between the contraction force (Fc) generated by carbon decomposition and the adhesion force (Fa) from the surrounding matrix. Once Fc exceeds Fa, layer-by-layer contraction occurs, triggering internal shell separation and the emergence of the characteristic triple-shell hollow structure. This Fc–Fa interplay is crucial in engineering the multi-shelled morphology [24].

Finally, vapor-phase sulfidation was employed to convert the HEO into a sulfide framework with Fe_4.2_Ni_4.8_S_8_ as the dominant crystalline phase. Importantly, the well-defined triple-shelled morphology was fully preserved through this transformation, evidencing the robustness of the sequential templating strategy in maintaining structural integrity.

**Figure 1:**
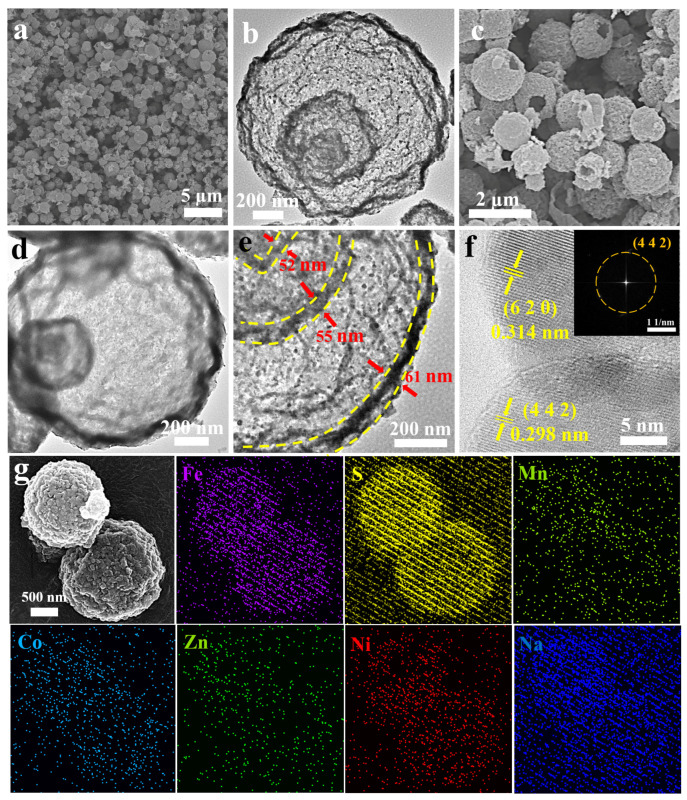
(**a**) SEM image of HEO. (**b**) TEM image of HEO. (**c**) SEM image of (NaFeZnCoNiMn)_9_S_8_. (**d**,**e**) TEM images of (NaFeZnCoNiMn)_9_S_8_ at various magnifications. (**f**) HRTEM image and FFT pattern of (NaFeZnCoNiMn)_9_S_8_. (**g**) EDS elemental mapping of (NaFeZnCoNiMn)_9_S_8_.

Comprehensive structural characterizations of HEO and (NaFeZnCoNiMn)_9_S_8_ are presented in Figure 1. The SEM image reveals that HEO exhibits a spherical morphology with diameters of approximately 1 μm (Figure 1a). TEM further confirms the presence of a multi-shelled structure within the spheres (Figure 1b). Following sulfurization production, (NaFeZnCoNiMn)_9_S_8_ retains the hollow triple-shelled architecture of the precursor, as evidenced by both SEM and TEM images (Figure 1c–e). High-magnification TEM images show that the shell thickness of (NaFeZnCoNiMn)_9_S_8_ is approximately 55 nm (Figure 1e). HRTEM image display distinct lattice fringes, with measured interplanar spacings of 0.314 nm and 0.298 nm corresponding to the (620) and (442) planes of Fe_4.2_Ni_4.8_S_8_, respectively. The SAED pattern further indicates the crystalline identity, showing bright spots that align well with the (442) crystalline planes (Figure 1f). Moreover, elemental mapping (Figure 1g) confirms the homogeneous distribution of Na, Fe, Zn, Co, Ni, Mn, and S throughout the (NaFeZnCoNiMn)_9_S_8_ matrix, affirming the successful synthesis of a chemically uniform high-entropy sulfide.

The phase composition and crystallinity of the synthesized samples were characterized by XRD. As shown in Figure S1, the diffraction peaks of the precursor HEO can be indexed to the spinel Fe_3_O_4_ phase (JCPDS No. 75-1610), confirming its successful formation. Figure S2 displays the XRD pattern of the final product (NaFeZnCoNiMn)_9_S_8_, with peaks corresponding well to the Fe_4.2_Ni_4.8_S_8_ phase (JCPDS No. 86-2279), indicating a successful phase transformation after sulfidation. Notably, as shown in Figure S2b, the XRD pattern of (NaFeZnCoNiMn)_9_S_8_ exhibits discernible peak shifts toward lower 2θ angles compared to the standard Fe_4.2_Ni_4.8_S_8_ reference card (PDF#86-2279). Specifically, the diffraction peaks corresponding to the (111), (200), (311), (222), (511), and (440) planes all show varying degrees of leftward shift. This phenomenon is attributed to lattice expansion induced by the incorporation of multiple metal cations with different ionic radii, further confirming the successful formation of a high-entropy phase [25].

**Figure 2:**
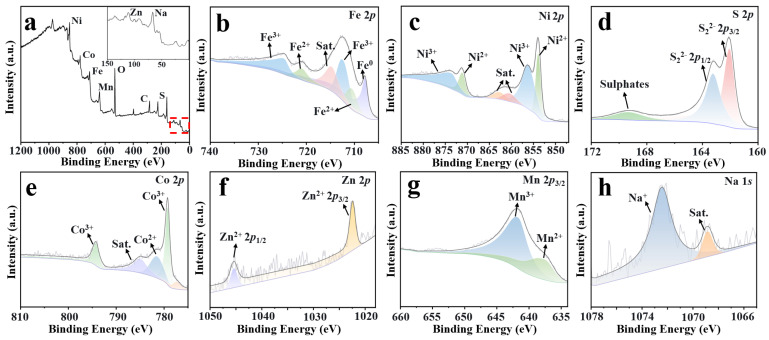
(**a**) XPS survey spectrum of (NaFeZnCoNiMn)_9_S_8_. High-resolution XPS spectra of (**b**) Fe 2*p*, (**c**) Ni 2*p*, (**d**) S 2*p*, (**e**) Co 2*p*, (**f**) Zn 2*p*, (**g**) Mn 2*p*, and (**h**) Na 1*s* of (NaFeZnCoNiMn)_9_S_8_.

The XPS survey spectrum of (NaFeZnCoNiMn)_9_S_8_, as shown in Figure 2a, reveals the presence of Ni, Co, Fe, Mn, Zn, Na, and S, verifying the incorporation of all intended elements, with the corresponding atomic percentages listed in Table S1. To further validate the bulk elemental composition, inductively coupled plasma optical emission spectroscopy (ICP-OES) analysis was also conducted, and the results are summarized in Table S2. The ICP-OES data confirm that the overall Fe:Ni ratio is approximately 9:10, consistent with the designed stoichiometry and complementing the surface-sensitive XPS findings. High-resolution XPS spectra (Figure 2b–h) further demonstrate the presence of multiple oxidation states, corroborating the multivalent nature of the transition-metal cations in the (NaFeZnCoNiMn)_9_S_8_ system. In the Fe 2*p* spectrum (Figure 2b), characteristic peaks at 710.9 eV and 721.1 eV are assigned to Fe^2+^ 2*p*_3/2_ and 2*p*_1/2_, while peaks at 712.7 eV and 724.9 eV correspond to Fe^3+^ 2*p*_3/2_ and 2*p*_1/2_, respectively, indicating the coexistence of Fe^2+^/Fe^3+^ oxidation states. For Ni 2*p* (Figure 2c), the observed peaks at 853.8/871.1 eV and 856.4/873.7 eV are attributed to Ni^2+^ and Ni^3+^ species, respectively. Similarly, Co 2*p* signals (Figure 2d) reveal peaks at 779.3 and 794.4 eV, corresponding to Co^3+^, along with a minor shoulder at 781.7 eV attributed to Co^2+^. In the Mn 2*p* region (Figure 2e), peaks at 637.8 eV and 641.9 eV suggest the co-presence of Mn^2+^ and Mn^3+^ species. Zn 2*p* peaks located at 1022.4 eV and 1045.5 eV (Figure 2f) are characteristic of divalent Zn^2+^, while the Na 1*s* peak at 1072.4 eV (Figure 2g) confirms the incorporation of monovalent Na^+^ into the lattice. Furthermore, the S 2*p* doublet centered at 162.1 eV and 163.3 eV (Figure 2h) corresponds to sulfide S^2−^ species, verifying the anionic framework of the sulfide matrix [26,27,28]. To demonstrate that the elemental composition is preserved during the sulfidation process, we also performed XPS analysis on the oxide precursor, which showed the presence of the same metal elements, including Na. The Na signal originates from the NaCl added during the hydrothermal synthesis step and remains detectable in the oxide precursor, as shown in Figure S3. The coexistence of these multivalent species suggests a complex electronic environment, where the diverse valence states are likely stabilized by the chemically disordered lattice and the varied local coordination environments inherent to high-entropy materials. This behavior is a hallmark of high-entropy sulfides, reflecting the strong configurational entropy and synergistic interactions among the multi-metallic components.

**Figure 3:**
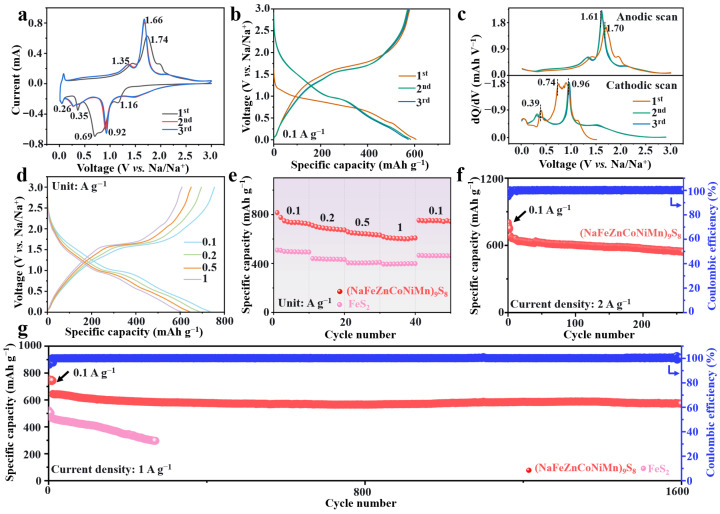
(**a**) CV curves of the (NaFeZnCoNiMn)_9_S_8_ anode. (**b**) GCD profiles at a current density of 0.1 A g^−1^. (**c**) Differential capacity (dQ/dV) plots of (NaFeZnCoNiMn)_9_S_8_ during the first three cycles. (**d**) Rate performance charge–discharge curves and (**e**) rate performance chart of (NaFeZnCoNiMn)_9_S_8_ electrode. Cycling performance of (NaFeZnCoNiMn)_9_S_8_ at current density of (**f**) 2 A g^−1^ and (**g**) 1 A g^−1^.

Electrochemical evaluations highlight the synergistic effects of molecular composition and microstructural design. Figure 3a shows the cyclic voltammetry (CV) curves of the first three cycles for the (NaFeZnCoNiMn)_9_S_8_. A broad reduction peak centered at 0.69 V is attributed to the formation of a solid electrolyte interphase [29], which vanishes in subsequent cycles and evolves into a sharper peak at 0.92 V. Meanwhile, anodic peaks at 1.35 and 1.74 V are associated with the reoxidation of reduced metallic species. The near-complete overlap of cathodic and anodic curves from the second cycle onward indicates excellent reversibility and electrochemical stability. The peak at 1.16 V corresponds to the solid-solution insertion of Na^+^ into the sulfide lattice, evidenced by continuous shift of peaks to lower angles. The peak at 0.35 V marks the conversion reaction onset, where new diffraction peaks emerge at 49.9° (Fe^0^), 41.2° (Ni^0^) and 52.3° (Mn^0^). The synchronous reduction in Fe/Ni/Mn (despite distinct theoretical potentials) reflects high-entropy-enabled redox synergy. During charging, the anodic peaks at 1.35 V and 1.74 V correlate the reformation of (NaFeZnCoNiMn)_9_S_8_ and the disappearance of the Na_2_S phase. A detailed mechanistic investigation will follow in the subsequent section.

It is noteworthy that, despite the presence of six metal elements in the (NaFeZnCoNiMn)_9_S_8_ system, only two pairs of distinct redox peaks are observed in the CV curves. This phenomenon can be attributed to the overlapping electrochemical potentials of the transition metal cations and their different degrees of electrochemical activity. The results indicate that, during the initial discharge, sequential conversion reactions of individual metal–sulfur (M–S) bonds occur, as reflected by the gradual disappearance and left-shift of characteristic peaks, and the emergence of signals attributed to reduced metallic species.

Therefore, the two observed redox pairs are likely the result of overlapping conversion reactions of multiple M–S bonds, with dominant contributions from electrochemically more active metal species. This behavior is in line with previous reports on high-entropy sulfides, where complex electronic interactions and varied local environments lead to selective redox participation among multiple cations.

The galvanostatic charge–discharge (GCD) curve at 0.1 A g^−1^ (Figure 3b) shows a notable initial irreversible capacity loss (743.6/789.9 mAh g^−1^), primarily attributed to the formation of the SEI, incomplete Na^+^ extraction during structural activation, and partial degradation of the electrolyte under reductive conditions. [30]. Notably, the presence of sodium in (NaFeZnCoNiMn)_9_S_8_ may reduce the amount of external Na^+^ required for SEI formation during the first cycle, thereby partially mitigating initial irreversible capacity loss and contributing to the improved ICE of 94.14%. From the second cycle onward, the electrode exhibits a high reversible capacity of approximately 582.8 mAh g^−1^, attributed to the synergistic effects of multicomponent composition and hollow triple-shell architecture. The differential capacity (dQ/dV) curves (Figure 3c) show well-defined voltage plateaus consistent with the CV profiles [31], reinforcing the stable redox behavior of the material. Despite the presence of multiple electrochemically active elements, only two pairs of redox peaks are observed, suggesting strong electronic hybridization and uniform atomic-scale distribution—hallmarks of high-entropy material design [32].

As shown in Figure 3d, the GCD curves collected at various current densities demonstrate the robust electrochemical performance of (NaFeZnCoNiMn)_9_S_8_. The rate performance was evaluated at current densities of 0.1, 0.2, 0.5, and 1.0 A g^−1^, delivering specific capacities of 735.3, 686.9, 643.2, and 603.1 mAh g^−1^, respectively, consistently outperforming FeS_2_. Notably, when the current density was returned to 0.1 A g^−1^, the capacity recovered to 749.6 mAh g^−1^, confirming the excellent rate reversibility (Figure 3e). At an ultra-high current density of 10 A g^−1^, the material maintained a reversible capacity of 285.2 mAh g^−1^ after 250 cycles with an ICE of 94.71% (Figure S4), exceeding the performance of many previously reported SIB anodes. After initial activation at lower currents over five cycles, the CE stabilized near 100%, indicating nearly complete Na^+^ extraction and reinsertion during each cycle [33].

The GCD curves of the (NaFeZnCoNiMn)_9_S_8_ electrode at a high current density of 2 A g^−1^ reveal nearly identical voltage plateaus even after more than 270 cycles. This consistency indirectly confirms the excellent cycling stability of the (NaFeZnCoNiMn)_9_S_8_ electrode under high current density (Figure S5) [34]. As shown in Figure 3f, the cycling performance corresponding to the GCD curves demonstrates that the electrode maintains an ideal discharge specific capacity of 531.9 mAh g^−1^ after 270 charge–discharge cycles at a high current density of 2 A g^−1^, with an ICE of 95.21%. Long-term cycling tests conducted at 1 A g^−1^ further reveal the exceptional durability of (NaFeZnCoNiMn)_9_S_8_, which retained 582.6 mAh g^−1^ after 1600 cycles, corresponding to a capacity retention of 92.4% and an ultra-low per-cycle decay rate of 0.06% (Figure 3g). Furthermore, the electrochemical performance of the (NaFeZnCoNiMn)_9_S_8_ electrode at high loading mass was also evaluated (Figure S6), and at loading mass of 3.5 mg cm^−2^, it can deliver the specific discharge capacity of 543.6 mAh g^−1^ after 60 cycles at 1 A g^−1^. In contrast, FeS_2_ exhibited rapid capacity fading after only 200 cycles, primarily due to its unoptimized morphology, which induced severe volume expansion and poor contact between the electrode and the current collector [35]. The enhanced performance of (NaFeZnCoNiMn)_9_S_8_ is attributed to the synergistic interactions among the multiple metal species in the high-entropy matrix, which collectively enhance electronic/ionic conductivity, facilitate fast reaction kinetics, and enable multivalent redox reactions that contribute to superior capacity [36,37].

**Figure 4:**
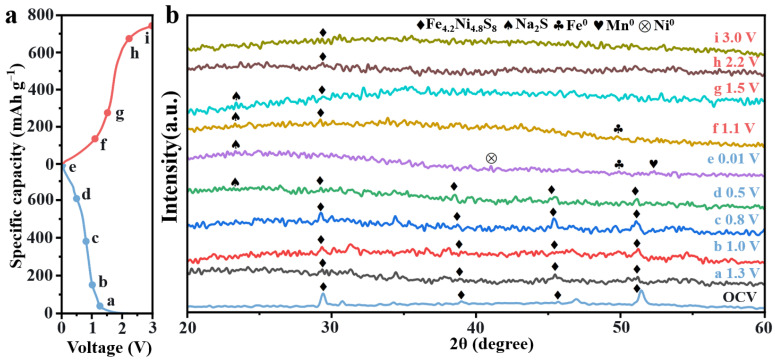
(**a**) Charge–discharge curves of the initial cycle at 0.1 A g^−1^ and (**b**) ex situ XRD patterns at different charge–discharge voltage states.

To gain deeper insight into the phase transformation behavior of (NaFeZnCoNiMn)_9_S_8_ during the initial charge–discharge cycle, ex situ XRD measurements were conducted on (NaFeZnCoNiMn)_9_S_8_ electrodes cycled in sodium–ion batteries (Figure 4). At the early stage of discharge, diffraction peaks at 29.4°, 39.0°, 46.9°, and 51.4° can be indexed to the (311), (331), (511), and (440) planes of Fe_4.2_Ni_4.8_S_8_, respectively. As the discharge progresses, the intensity of these peaks gradually diminishes and shifts toward lower angles, indicating progressive Na^+^ insertion into the lattice.

Meanwhile, a diffraction peak at 23.5° emerges, corresponding to Na_2_S. When the discharge voltage reaches 0.01 V, weak peaks appear at 41.2°, 49.9°, and 52.3°, which are attributed to the formation of metallic Ni^0^, Fe^0^, and Mn^0^, respectively. These peaks exhibit low intensity likely due to the nanocrystalline or partially amorphous nature of the metallic phases.

During the subsequent desodiation process, the intensity of the Na_2_S peak gradually decreases, and the characteristic peak of (NaFeZnCoNiMn)_9_S_8_ reappears at 29.5°, suggesting the partial reformation of the original structure. This observation highlights the unique structural stability of the high-entropy material. The conversion reaction is likely favored within the high-entropy matrix, which facilitates the redistribution of cations back into their original lattice positions, thereby maintaining the integrity of the high-entropy sulfide-derived framework [38].

These findings further validate the successful synthesis and functional performance of the high-entropy (NaFeZnCoNiMn)_9_S_8_ electrode material. Based on the above observations, the main electrochemical reactions during the Na^+^ insertion and extraction processes can be summarized as follows:

Fe_4.2_Ni_4.8_S_8_ + x Na^+^ + x e^−^ ⇌ Na_x_Fe_4.2_Ni_4.8_S_8_ (1)

Na_x_Fe_4.2_Ni_4.8_S_8_ + (16 − x) Na^+^ + (16 − x) e^−^ ⇌ 4.2 Fe^0^ + 4.8 Ni^0^ + 8Na_2_S (2)

**Figure 5:**
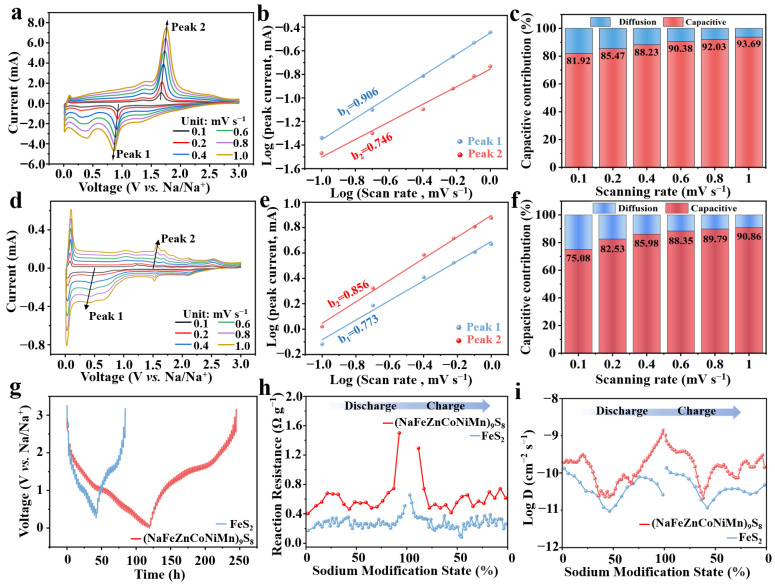
(**a**) CV curves of (NaFeZnCoNiMn)_9_S_8_ at different scan rates. (**b**) log(*i*) vs. log(*v*) fitting curve of (NaFeZnCoNiMn)_9_S_8_. (**c**) Pseudocapacitive contribution of (NaFeZnCoNiMn)_9_S_8_ at different scan rates. (**d**) CV curves of FeS_2_ at different scan rates. (**e**) log(*i*) vs. log(*v*) fitting curve of FeS_2_. (**f**) Pseudocapacitive contribution of FeS_2_ at different scan rates. (**g**) GITT curve of (NaFeZnCoNiMn)_9_S_8_ anode. (**h**) Calculated reaction impedance. (**i**) Changes in *D*_Na^+^_ during the charge and discharge process.

To elucidate the reaction kinetics of (NaFeZnCoNiMn)_9_S_8_ and FeS_2_ as anode materials for SIBs, cyclic voltammetry (CV) was conducted at various scan rates (Figure 5a,d). The redox peaks for both materials exhibited systematic shifts, indicating consistent electrochemical behavior. The pseudocapacitive contribution was quantitatively assessed using the equations *i = av*^b^ and log (*i*) *=* log (*a*) + *b* log (*v*) [39], where the slope *b* distinguishes between diffusion-controlled (*b* = 0.5) and capacitive-controlled (*b* = 1.0) processes. The calculated *b* values for (NaFeZnCoNiMn)_9_S_8_ were 0.906 and 0.746, exceeding those of FeS_2_(0.856 and 0.773), suggesting a greater pseudocapacitive contribution in the high-entropy sulfide (Figure 5b,e).

Furthermore, the pseudocapacitive fraction increased with scan rate. The pseudocapacitive contributions of (NaFeZnCoNiMn)_9_S_8_ and FeS_2_ reached 93.69% and 90.86% at 1 mV s^−1^, respectively (Figure 5c,f). These findings highlight the effectiveness of compositional and structural engineering in optimizing sodium storage performance. Specifically, the multi-element doping facilitates faster Na^+^ transport, while the multi-shell architecture significantly reduces diffusion pathways, collectively enhancing pseudocapacitive behavior and rate capability. To further elucidate the Na^+^ transport kinetics of (NaFeZnCoNiMn)_9_S_8_ during the multi-step Na^+^ insertion/desertion process, galvanostatic intermittent titration technique (GITT) measurements were conducted on both (NaFeZnCoNiMn)_9_S_8_ and FeS_2_ (Figure 5g). The total interfacial resistance during charge/discharge was extracted from the GITT profiles (Figure 5h), revealing that (NaFeZnCoNiMn)_9_S_8_ exhibits a significantly lower interfacial resistance compared to FeS_2_, indicative of improved charge transfer kinetics.

Furthermore, the sodium–ion diffusion coefficients (*D*_Na^+^_) were calculated based on the following equation: *D*_Na^+^_ = 4/*πt*(*mV*_m_/*MA*)^2^ (*ΔE*_s_/*ΔE*_τ_)^2^, where *τ* is the pulse duration, *m*, *V*_m_, and *M* represent the mass, molar volume, and molar mass of the active material, respectively; *A* denotes the electrode area; *ΔE*_s_ is the steady state potential change before and after the current pulse; and *ΔE*_τ_ is the voltage change during the constant current step [40]. As shown in Figure 5i, (NaFeZnCoNiMn)_9_S_8_ demonstrates markedly enhanced Na^+^ diffusivity, with log *D*_Na^+^_ values ranging from 10^−8.5^ to 10^−10.5^ cm^2^ s^−1^, substantially higher than those of MoS_2_ (10^−10.1^ to 10^−11.2^ cm^2^ s^−1^). These results highlight the efficacy of high-entropy engineering in accelerating sodium–ion diffusion by tailoring electronic structure and defect chemistry, which is critical for improving the overall reaction kinetics in SIBs.

The excellent fast-charging performance of (NaFeZnCoNiMn)_9_S_8_ can be ascribed to a combination of high-entropy effects, optimized interfacial kinetics, and significant pseudocapacitive contributions. First, the incorporation of multiple transition-metal cations induces pronounced lattice distortion and configurational disorder, which promotes Na^+^ transport through enhanced ionic conductivity. Simultaneously, the uniform distribution of redox-active species allows for multiple reversible redox reactions to occur in parallel, facilitating rapid electron exchange and improving charge transport kinetics. Second, EIS and GITT analyses demonstrate reduced charge-transfer resistance and elevated sodium-ion diffusion coefficients for (NaFeZnCoNiMn)_9_S_8_ relative to FeS_2_, confirming superior interfacial ion transport. Third, CV analysis at varying scan rates (Figure 5a–c) reveals that pseudocapacitive processes dominate the charge storage behavior, with a pseudocapacitive contribution reaching 93.69% at 1 mV s^−1^. This fast surface-controlled mechanism is particularly favorable under high-rate cycling conditions.

Collectively, these features confirm the outstanding fast-charge capability of the (NaFeZnCoNiMn)_9_S_8_ anode material, as a result of synergistic compositional and structural engineering.

**Figure 6:**
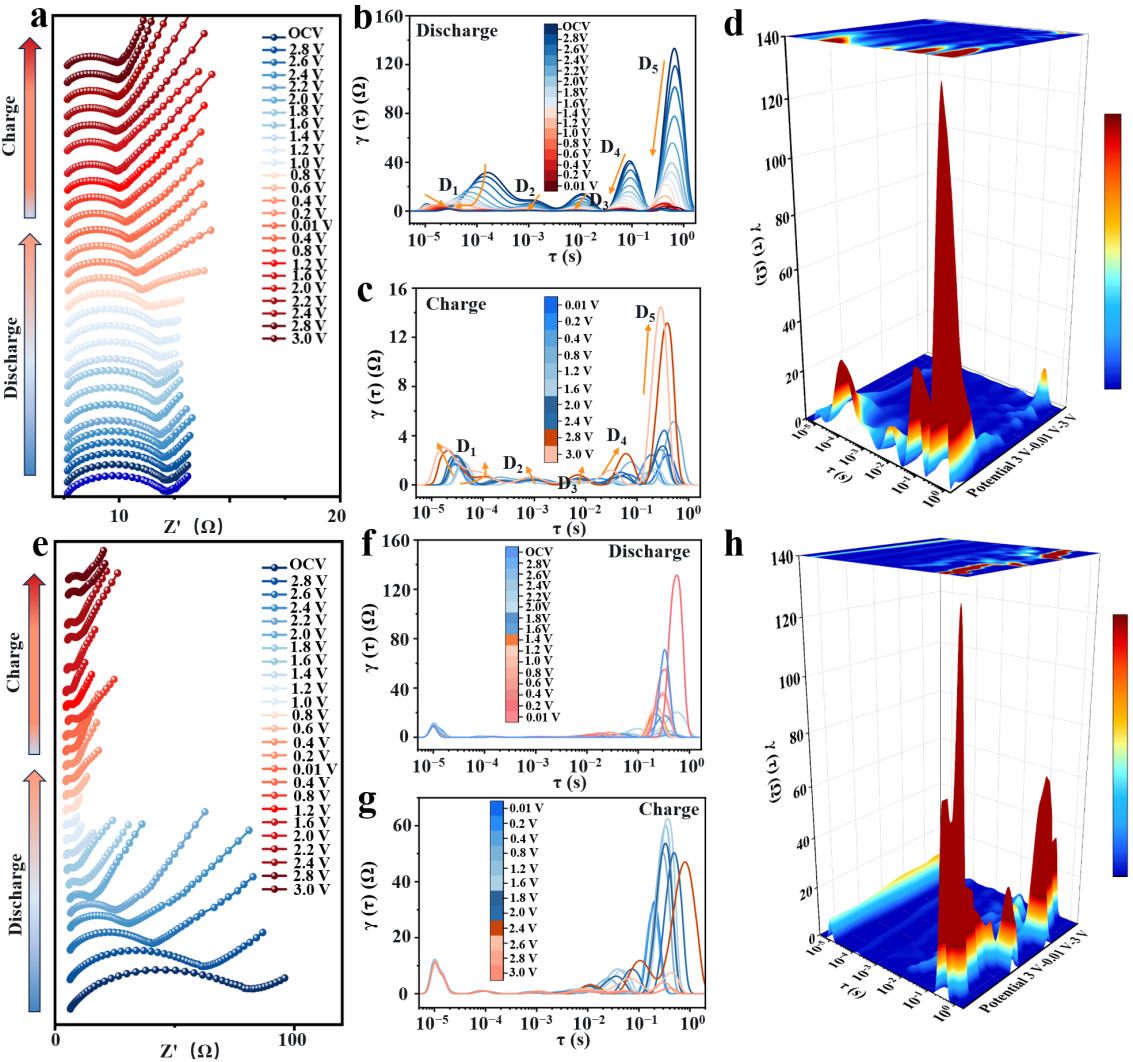
(**a**) In situ EIS spectra of the (NaFeZnCoNiMn)_9_S_8_ anode during the first electrochemical cycle. (**b**,**c**) DRT profiles derived from the in situ EIS data at various voltages. (**d**) 3D contour plot of the DRT fitting results for (NaFeZnCoNiMn)_9_S_8_. (**e**) In situ EIS spectra of the FeS_2_ anode during the first electrochemical cycle. (**f**,**g**) DRT profiles derived from the in situ EIS data at various voltages for FeS_2_. (**h**) 3D contour plot of the DRT fitting results for FeS_2_.

To gain further insight into the dynamic interfacial processes of (NaFeZnCoNiMn)_9_S_8_ during charge–discharge cycles, in situ electrochemical impedance spectroscopy (EIS) was conducted during the initial cycle (Figure 6a), and the resulting data at various voltages were deconvoluted via distribution of relaxation times (DRT) analysis [41]. This approach provides a time-resolved perspective on impedance evolution at the electrode–electrolyte interface [42]. As shown in Figure 6b,f, five distinct peaks were identified across a time constant (*τ*) range of 10^−5^ to 1 s, each representing a specific electrochemical process.

The D_1_ peak is attributed to the contact resistance (*R*_s_) of the electrode material. For (NaFeZnCoNiMn)_9_S_8_, *R*_s_ initially decreases and then stabilizes during discharge, whereas FeS_2_ maintains a persistently elevated *R*_s_ of ~12.6 Ω throughout the cycle (Figure 6e). This reduction in contact resistance is ascribed to the hollow triple-shelled architecture of (NaFeZnCoNiMn)_9_S_8_, which improves electrolyte penetration and interfacial wetting. The D_2_ and D_3_ peaks correspond to the SEI formation and its associated ionic transport resistance (*R*_SEI_). Notably, for (NaFeZnCoNiMn)_9_S_8_, both peaks diminish significantly during discharge and reach minimum values of 0.36~0.63 Ω at 0.01 V, which are lower than those observed for FeS_2_ (0.56~0.76 Ω at the same voltage). These findings indicate the generation of a more homogeneous and conductive SEI layer in the high-entropy material, likely stemming from the synergistic interactions among the multiple transition metals in the lattice [43].

The D_4_ peak is attributed to the charge-transfer resistance (*R*_ct_), which reflects the interfacial electrochemical reaction kinetics. During discharge, (NaFeZnCoNiMn)_9_S_8_ exhibits a pronounced reduction in *R*_ct_, in contrast to FeS_2_, which shows irregular and poorly correlated changes in peak intensity, suggesting sluggish and unstable interfacial reactions. Remarkably, during the charging process, the *R*_ct_ for (NaFeZnCoNiMn)_9_S_8_ was significantly lower than that for FeS_2_, throughout the discharging/charging cycle, highlighting the superior charge-transfer efficiency imparted by the high-entropy configuration (Figure 6c,g). The D_5_ peak corresponds to the Warburg-type diffusion resistance (*W*_diff_), which is associated with ion transport within the electrode. (NaFeZnCoNiMn)_9_S_8_ displays a distinct decrease followed by a gradual increase in *W*_diff_ throughout the full charge–discharge cycle, reflecting a highly reversible electrochemical process. More importantly, its diffusion resistance during discharge remains consistently lower than that of FeS_2_ (Figure 6d,h), underscoring the effectiveness of rational compositional tuning and multi-shelled architecture in shortening Na^+^ diffusion pathways and mitigating ion transport limitations [44,45].

In summary, the synergistic combination of high-entropy composition and hierarchical triple-shelled architecture in (NaFeZnCoNiMn)_9_S_8_ significantly enhances interfacial charge transfer and facilitates rapid Na^+^ migration. This integrated design strategy effectively addresses the inherently sluggish ion transport kinetics that traditionally hinder the electrochemical performance of transition metal sulfide-based anodes.

**Figure 7:**
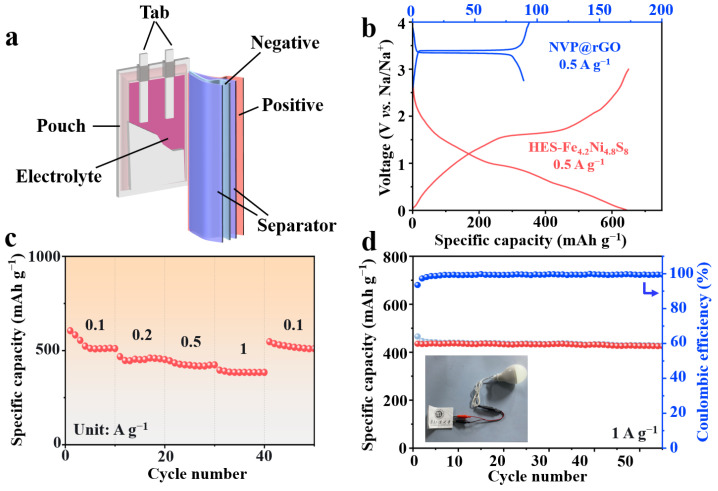
(**a**) Schematic illustration of the configuration of the soft-packed full cell. (**b**) GCD profiles of the NVP@rGO cathode and (NaFeZnCoNiMn)_9_S_8_ anode in half-cell configurations at a current density of 0.5 A g^−1^. (**c**) Rate performance chart of the assembled NVP@rGO//(NaFeZnCoNiMn)_9_S_8_ coin-type full cell. (**d**) Cycling performance of the assembled NVP@rGO//(NaFeZnCoNiMn)_9_S_8_ coin-type full cell at 1 A g^−1^ and demonstrating the capability of the soft-packed device to power a commercial household LED lamp.

To further validate the practical applicability of (NaFeZnCoNiMn)_9_S_8_ as an anode material for SIBs, a full cell was assembled using (NaFeZnCoNiMn)_9_S_8_ as the anode and NVP@rGO as the cathode, denoted as NVP@rGO//(NaFeZnCoNiMn)_9_S_8_. The configuration of the soft-packed full cell is illustrated in Figure 7a. Based on the GCD profiles (Figure 7b), an optimal voltage window of 1.0~3.5 V was established [46,47]. Additionally, the full cell demonstrated superior rate capabilities (Figure 7c), delivering rate capacities of 509.9, 446.1, 418.8, and 383.6 mAh g^−1^ at 0.1, 0.2, 0.5, and 1 A g^−1^, respectively. The assembled coin-type full cell exhibited a high reversible capacity of 425.3 mAh g^−1^ after 55 cycles at a current density of 1 A g^−1^ (Figure 7d). Moreover, the soft-packed device successfully powered a household LED lamp, underscoring its promise for real-world energy storage applications.

## 3. Conclusions

A hollow triple-shelled high-entropy sulfide, (NaFeZnCoNiMn)_9_S_8_, was successfully synthesized via a modified sequential templating approach. This distinctive multi-shelled architecture, combined with a high-entropy configuration, effectively shortens Na^+^ diffusion pathways and accommodates volume changes during cycling. Electrochemical analysis revealed an outstanding ICE of 94.14% at current densities of 0.1 A g^−1^, alongside high specific capacities of 735.3, 686.9, 643.2, and 603.1 mAh g^−1^ at current densities of 0.1, 0.2, 0.5, and 1.0 A g^−1^, respectively. Notably, when the current density was returned to 0.1 A g^−1^, the capacity recovered to 749.6 mAh g^−1^, highlighting excellent rate performance. Even at an ultrahigh current density of 10 A g^−1^, a reversible capacity of 285.2 mAh g^−1^ was retained after 250 cycles. Long-term testing at 1 A g^−1^ delivered a capacity of 582.6 mAh g^−1^ after 1600 cycles, with a retention of 90.4% and a minimal per-cycle decay of just 0.06%. In a full-cell configuration (NVP@rGO//(NaFeZnCoNiMn)_9_S_8_), a reversible capacity of 425.3 mAh g^−1^ was maintained over 55 cycles. These remarkable electrochemical properties arise from the synergistic advantages of the hierarchical shell structure, enhanced charge transfer dynamics, and multivalent cationic interactions within the high-entropy matrix. This study provides a compelling strategy for the design of next-generation high-performance SIBs anode materials based on multi-shelled high-entropy architectures.

## Data Availability

Data will be made available upon request.

## Supplementary Materials

The following supporting information can be downloaded at: https://www.mdpi.com/article/10.3390/nano15120881/s1, Figure S1: XRD patterns of HEO; Figure S2: (a) XRD patterns of (NaFeZnCoNiMn)_9_S_8_. (b) A magnified view of diffraction peak from (a); Figure S3: (a) XPS survey spectrum of HEO. High-resolution XPS spectra of (b) Fe 2*p*, (c) Ni 2*p*, (d) S 2*p*, (e) Co 2*p*, (f) Zn 2*p*, (g) Mn 2*p* and (h) Na 1*s* of HEO; Figure S4: Cycling performance of (NaFeZnCoNiMn)_9_S_8_ at a current density of 10 A g^−1^; Figure S5: GCD curves at a current density of 2 A g^−1^; Figure S6: The cycling performance of (NaFeZnCoNiMn)_9_S_8_ at high loading mass; Table S1: Surface elemental composition derived from XPS survey spectra; Table S2: The ICP-OES results of (NaFeZnCoNiMn)_9_S_8_.
