# Supplementary material for: High-Entropy Sulfide Nanoarchitectures with Triple-Shelled Hollow Design for Durable Sodium–Ion Batteries
Source: Nanomaterials (Basel). 2025 Jun 7;15(12):881. doi: 10.3390/nano15120881 (PMC12196503; doi:10.3390/nano15120881)
Supplement: Supplementary file 1 [file nanomaterials-15-00881-s001.zip › nanomaterials-3674970-supplementary.pdf]

Supporting Information

High-Entropy Sulfide Nanoarchitectures with Triple-Shelled Hollow Design for Durable Sodium–Ion Batteries

Mingyang Chen [1], Yan Liu [2], Zhenchun Fang [1], Yinan Wang [1], Shaonan Gu [1,*] and Guowei Zhou [1,*]

[1] Key Laboratory of Fine Chemicals in Universities of Shandong, Jinan Engineering Laboratory for Multi-Scale Functional Materials, School of Chemistry and Chemical Engineering, Qilu University of Technology (Shandong Academy of Sciences), Jinan 250353, China;
[2] Zaozhuang Vocational College of Science & Technology, Tengzhou 277500, China;
* Correspondence: sngu@qlu.edu.cn (S.G.); gwzhou@qlu.edu.cn (G.Z.)

Materials and Methods

Chemical

Thiourea (CH_4N_2S), ferric chloride hexahydrate ($FeCl_3{\cdot}6H_2O$), cobalt chloride hexahydrate ($CoCl_2{\cdot}6H_2O$), manganese chloride tetrahydrate ($MnCl_2{\cdot}4H_2O$), zinc chloride dihydrate ($ZnCl_2{\cdot}2H_2O$), nickel chloride hexahydrate ($NiCl_2{\cdot}6H_2O$), iron disulfide (FeS_2), sodium chloride (NaCl), and anhydrous ethanol were all purchased from Sinopharm Chemical Reagent Co. Ltd, China.

Synthesis of HEO

First, 39 g of sucrose was dissolved in 50 mL of deionized water and transferred into a Teflon-lined autoclave for a hydrothermal reaction. The resulting product was washed three times with deionized water to obtain carbon spheres. Subsequently, 1.36 g $FeCl_3{\cdot}6H_2O$, 0.55 g $CoCl_2{\cdot}6H_2O$, 0.372 g $ZnCl_2{\cdot}2H_2O$, 0.622 g $MnCl_2{\cdot}4H_2O$, 0.727 g $NiCl_2{\cdot}6H_2O$, 0.223 g NaCl, and sucrose were dissolved in deionized water and mixed. The resulting solution was then subjected to a hydrothermal reaction in an autoclave. The obtained products were separated and washed alternately with deionized water and ethanol. The collected precursors were then calcined in a muffle furnace at 450 °C with a heating rate of 3 °C min^{-1} to obtain the final HEO product.

Synthesis of $(NaFeZnCoNiMn)_9S_8$

$(NaFeZnCoNiMn)_9S_8$ was synthesized by sulfidation of HEO in a tubular furnace. The process was conducted at 350 °C under an Ar/H_2 (95:5, v/v) atmosphere with a heating rate of 5 °C min^{-1}. Thiourea and HEO were separately placed upstream and downstream within the same ceramic boat to ensure directional vapor transport during the reaction. The resulting $(NaFeZnCoNiMn)_9S_8$ retained the distinctive hollow triple-shelled architecture inherited from the HEO precursor.

Synthesis of $Na_3V_2(PO_4)_3$@rGO

Na_2CO_3, $V(C_5H_7O_2)_3$ and $NH_4H_2PO_4$ were added to 40 mL of deionized water in the mass ratio (1.5:2:3) and then stirred to obtain a homogeneous solution. The solution was transferred to a 100 mL Teflon-lined stainless steel autoclave and heated at 180 °C for 12 h. Then, 10 mL of GO solution was added with stirring and then freeze-drying. The $Na_3V_2(PO_4)_3$@rGO powder was obtained by annealing above processor at 750 °C for 8 h in an Ar/H_2 (95:5 V/V) atmosphere.

Materials characterization

X-ray powder diffraction (XRD, BRUKER D8 ADVANCE) via Cu Kα (λ = 0.15406 nm) was used to characterize the phase and crystal structure. The Raman spectra were obtained using the Renishaw Via9 Raman microscope with a 633 nm laser. Transmission electron microscopy (TEM, JEOL, JEM–2100), scanning electron microscopy (SEM, Hitachi S-4800), high-resolution transmission electron microscopy (HRTEM, JEOL, JEM-2100F) and energy dispersive X-ray

spectroscopy (EDS) were tested to investigate the morphology and microstructures. X-ray photoelectron spectroscopy (XPS, PerkinElmer PHI 5300) was used to analyze the surface composition and elemental valence states.

Electrochemical measurements

The Na^+ storage performances of $(NaFeZnCoNiMn)_9S_8$ were tested by assembling CR2032 coin cells. The active material, Super P and sodium carboxymethyl cellulose (CMC) were mixed with water in a weight ratio of 7:2:1 and ball milled to obtain a slurry. The slurry was then applied to the copper foil and dried. The copper foil was cut into circular sheets with a diameter of 12 mm, where the mass loading of active material was 1.5 mg cm^{-2}. Metal Na was used as the counter and reference electrode, glass fiber membrane (Whatman GF/F) was used as the separator, and 1 M $NaPF_6$ dissolved in diethylene glycol dimethyl ether (DEGDME) was used as the electrolyte. Constant-current charge-discharge tests (GCD) and constant-current intermittent titration tests (GITT) were performed on LAND battery test system (CT2001A, China). The electrochemical workstation CHI 1000C was used for cyclic voltammetry (CV) testing. PARSTAT 4000 was used for electrochemical impedance spectroscopy (EIS) testing. To make the cathode electrode, $Na_3V_2(PO_4)_3$@rGO, Super P and polyvinylidene fluoride (PVDF) were mixed with N-methyl-2-pyrrolidone (NMP) in a mass ratio of 8:1:1 to form a slurry, which was then coated onto Al foil. The sodium ion full-cells were assembled with $(NaFeZnCoNiMn)_9S_8$ anode and $Na_3V_2(PO_4)_3$@rGO(NVP@rGO) cathode, and the capacity ratio of the FeS/MoS_2@NC and $Na_3V_2(PO_4)_3$@rGO is about 1: (1.2–1.5).

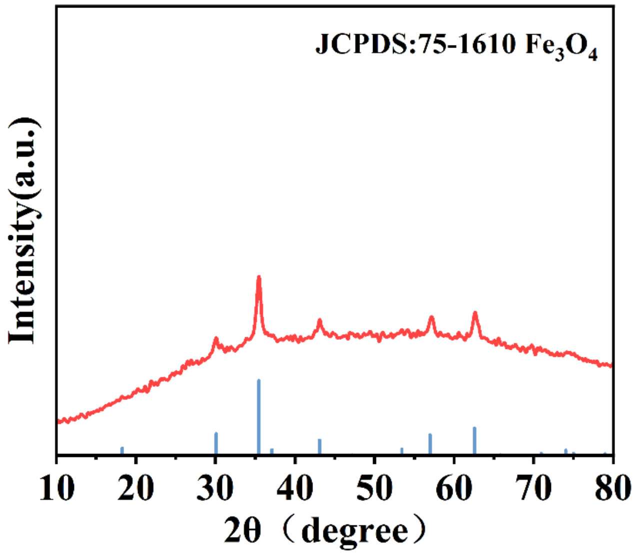

Figure S1. XRD patterns of HEO.

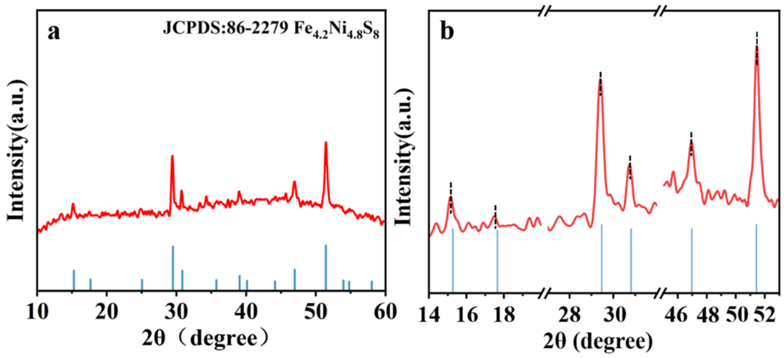

Figure S2. (a) XRD patterns of $(NaFeZnCoNiMn)_9S_8$. **(b)** A magnified view of diffraction peak from (a).

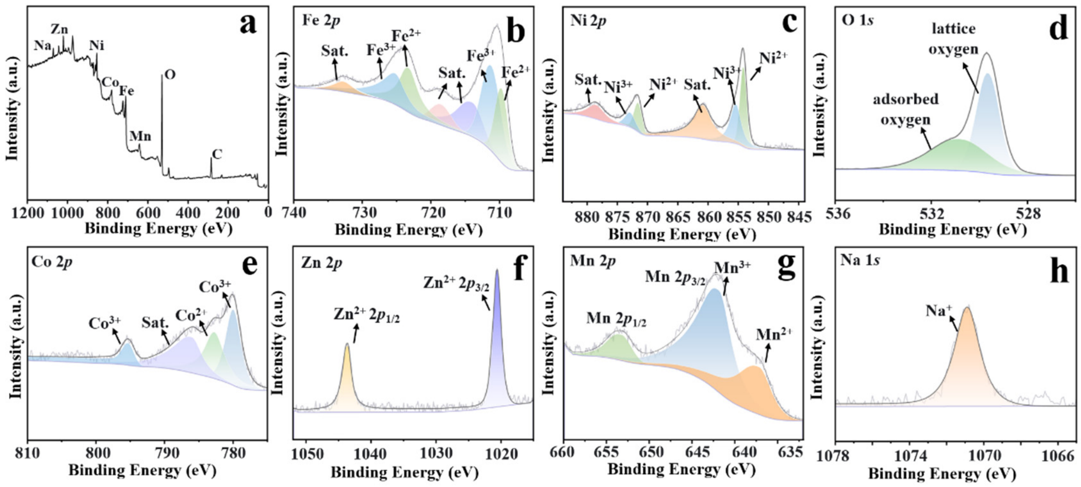

Figure S3. (a) XPS survey spectrum of HEO. High-resolution XPS spectra of **(b)** Fe 2*p*, **(c)** Ni 2*p*, **(d)** S 2*p*, **(e)** Co 2*p*, **(f)** Zn 2*p*, **(g)** Mn 2*p* and **(h)** Na 1*s* of HEO. .

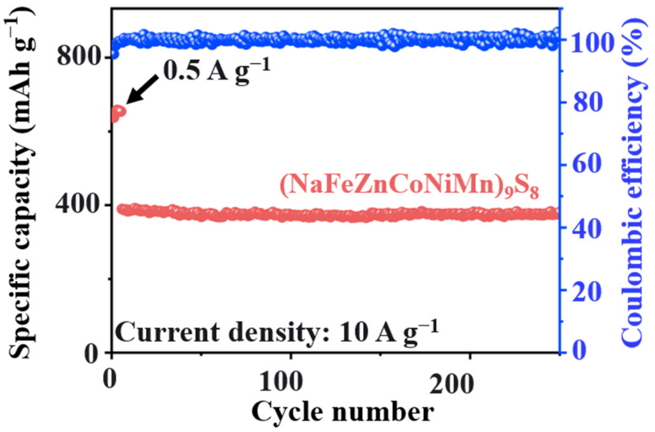

Figure S4. Cycling performance of $(NaFeZnCoNiMn)_9S_8$ at a current density of 10 A g^{-1}.

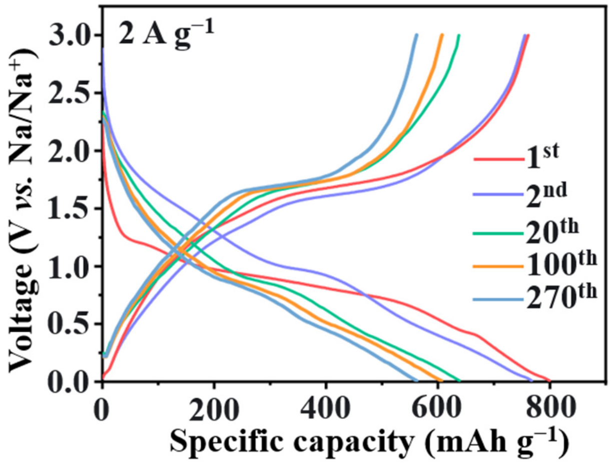

Figure S5. GCD curves at a current density of 2 A g^{-1}.

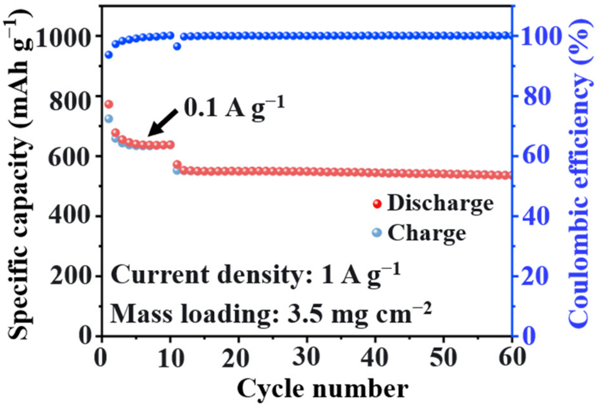

Figure S6. The cycling performance of $(NaFeZnCoNiMn)_9S_8$ at high loading mass.

Table S1. Surface elemental composition derived from XPS survey spectra.

Elements	Core level	Atomic percent (%)
Fe	2*p*	14.40
Ni	2*p*	5.41
S	2*p*	34.9
Co	2*p*	3.89
Zn	2*p*	1.46
Mn	$2p_{3/2}$	3.29
Na	1*s*	2.50
C	1*s*	31.15

Table S2. The ICP-OES results of $(NaFeZnCoNiMn)_9S_8$.

Elements	Concentration (ppb)
Na	294.32
Fe	2079.76
Zn	350.46
Co	1365.56
Ni	2293.61
Mn	202.58
